# ELBARA II, an L-Band Radiometer System for Soil Moisture Research

**DOI:** 10.3390/s100100584

**Published:** 2009-01-13

**Authors:** Mike Schwank, Andreas Wiesmann, Charles Werner, Christian Mätzler, Daniel Weber, Axel Murk, Ingo Völksch, Urs Wegmüller

**Affiliations:** 1 Gamma Remote Sensing AG, Worbstrasse 225, 3073 Gümligen, Switzerland; E-Mails: wiesmann@gamma-rs.ch (A.W.); cw@gamma-rs.ch (C.W.); wegmuller@gamma-rs.ch (U.W.); 2 Swiss Federal Research Institute WSL, Zürcherstrasse 111, 8903 Birmensdorf, Switzerland; E-Mail: ingo.voelksch@wsl.ch (I.V.); 3 Institute of Applied Physics, University Bern, Sidlerstr. 31, 3012 Bern, Switzerland; E-Mails: christian.matzler@iap.unibe.ch (C.M.); daniel.weber@iap.unibe.ch (D.W.); axel.murk@mw.iap.unibe.ch (A.M.)

**Keywords:** microwave, radiometer, remote sensing, Soil Moisture and Ocean Salinity Mission (SMOS)

## Abstract

L-band (1–2 GHz) microwave radiometry is a remote sensing technique that can be used to monitor soil moisture, and is deployed in the Soil Moisture and Ocean Salinity (SMOS) Mission of the European Space Agency (ESA). Performing ground-based radiometer campaigns before launch, during the commissioning phase and during the operative SMOS mission is important for validating the satellite data and for the further improvement of the radiative transfer models used in the soil-moisture retrieval algorithms. To address these needs, three identical L-band radiometer systems were ordered by ESA. They rely on the proven architecture of the ETH L-Band radiometer for soil moisture research (ELBARA) with major improvements in the microwave electronics, the internal calibration sources, the data acquisition, the user interface, and the mechanics. The purpose of this paper is to describe the design of the instruments and the main characteristics that are relevant for the user.

## 1. Introduction

Heat fluxes through the terrestrial surface layer are major drivers of climate. For land areas with sparse or no vegetation, the quantities involved in this energy exchange are fundamentally linked with the moisture in the soil surface. Techniques for monitoring the surface moisture on the spatial scales relevant for climate and meteorological research are therefore of particular interest [1–5].

Almost 25 years ago, it was suggested that soil moisture could be retrieved from remotely sensed thermal radiance received with an L-band radiometer [6,7]. Today L-band radiometry is one of the most promising approaches for remote soil-moisture retrieval since: (i) the atmosphere and clouds are almost transparent, thus allowing for all-weather measurements; (ii) the impact of vegetation canopies and surface roughness is less distinct compared with passive measurements at higher frequencies and active remote sensing techniques (radar); (iii) solar radiation affects radiometer measurements at the L-band only insignificantly, which allows for measurements at any time of the day; (iv) the 1,400−1,427 MHz frequency band is protected, which means that distortions of thermal radiance due to man-made radio frequency interferences (RFI) are minimized. However, in the past years several field experiments performed in Europe have shown that RFI is present even in the protected part of the L-band.

**Figure 1.**
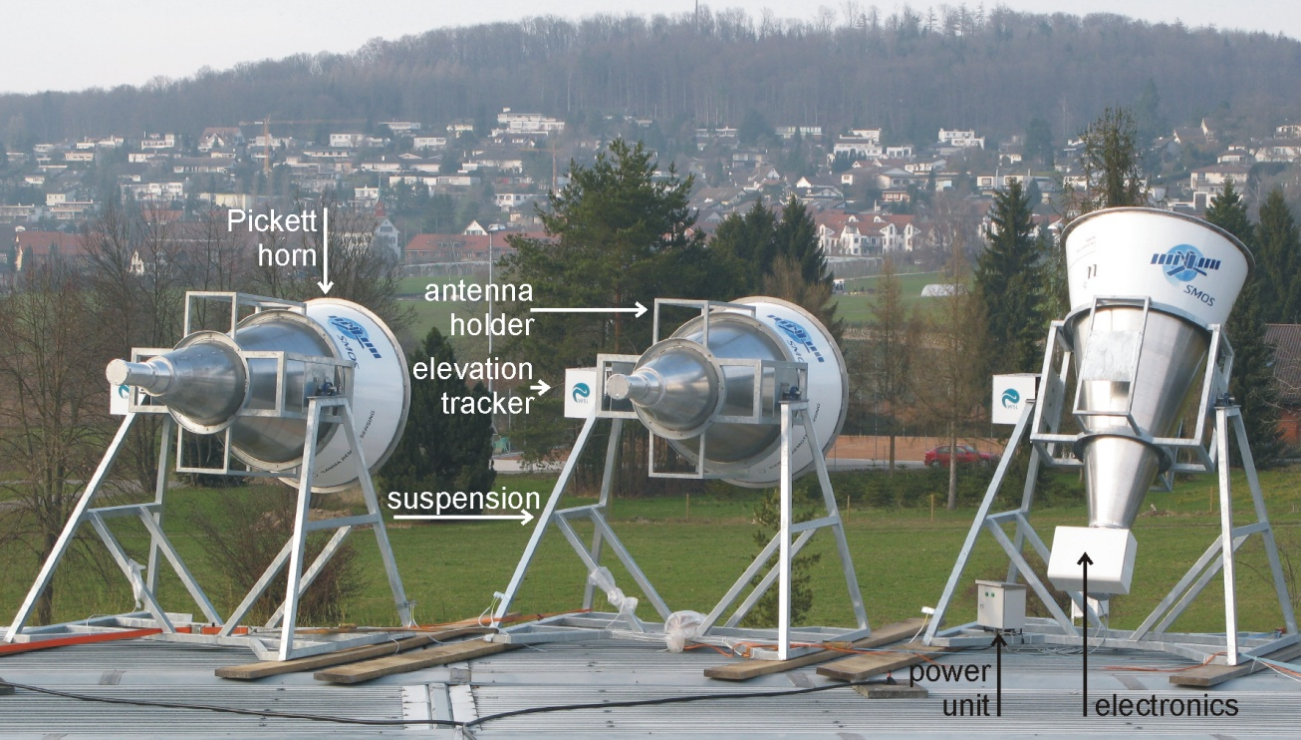
The ELBARA II systems mounted above the test site at the Swiss Federal Research Institute WSL, with the radiometer electronics and the power unit installed in the unit on the right.

During the calibration and validation activities associated with ESA’s SMOS mission [8] it turned out that further ground-based passive L-band experiments would be indispensable for the commissioning and the operative phase of the mission. To address this need, the three identical radiometers ELBARA II depicted in Figure 1 were built by Gamma Remote Sensing (Gümligen, Switzerland) as ordered by the ESTEC, in the framework of the contract ESTEC 21013/07/NL/FF “L-band Radiometer Systems to be deployed for SMOS Cal/Val Purposes”.

In the following paragraphs, we describe some basics of microwave radiometry, the requirements of the SMOS mission, and the corresponding research activities. The design and the main characteristics of the ELBARA II instruments are described in Sections 2 and 3. The Appendix contains a list of the abbreviations used and the specifications of the electronic components used in the radiometer design.

### 1.1. Measurement Principle

Microwave radiometry is a passive remote-sensing technique that measures thermal radiation. The radiance *T*_B_*^p^* emitted from a terrestrial surface at horizontal (*p* = H) or vertical (*p* = V) polarization depends on the surface temperature *T*_S_, and on the surface reflectivity *R^p^*. The latter can be used as a proxy for the remote retrieval of soil moisture or sea salinity.

In the microwave range, the Planck function of thermal radiation is linear with the absolute temperature. In this so-called Rayleigh-Jeans approximation, the upwelling brightness temperature of the emitted radiation above a surface is *T*_S_·(1 − *R^p^*). Since downwelling radiation *T*_sky_ also contributes to the observed radiation by the fraction reflected at the surface, the total radiation *T*_B_*^p^* received by a radiometer oriented towards the surface can be expressed by:

(1) $$T_{B}^{p}=(1-R^{p})T_{S}+R^{p}T_{\text{sky}}$$

The value of *T*_sky_ is determined by the cosmic background temperature of ≈2.7 K and enhanced by an atmospheric contribution. At 1.4 GHz, this enhancement is almost constant, leading to 4 K < *T*_sky_ < 5 K. Since the terrestrial surface temperature is much larger than *T*_sky_, the brightness temperature *T*_B_*^p^* has a strong sensitivity to *R^p^*.

The sensitivity of *T*_B_*^p^* with volumetric soil water content *WC* [m^3^ m^−3^] is established through *R^p^*, being dependent on the relative dielectric constant. The latter is a strong function of *WC* due to the marked contrast between the permittivity of free water (≈80) and dry soil (≈3 to 5). This allows the soil surface-water content to be determined from its reflectivity by applying dielectric mixing (e.g., [9–11]) and radiative transfer models. Typically, *T*_B_*^p^* of a very dry bare soil can be 150 K higher than for the same soil in the saturated moisture state.

Two different soil-depth ranges are of relevance: First, *T*_S_ represents an effective soil temperature averaged over the emission depth of the microwave radiation in the soil [12]. For a dry soil this can be 1 m or even more at 1.4 GHz, whereas for a wet soil the emission depth may be as little as a few centimeters [13]. Second, *R^p^* represents an effective surface reflectivity as a result of the dielectric transition from air to bulk soil with a more or less constant permittivity. In the simplest case of a homogeneous soil with a flat surface, the Fresnel equations [14] can be used to represent *R^p^* at polarization *p* = H, V and for a certain observation angle. At 1.4 GHz, a requirement for applying the Fresnel equations is a transition depth of <1 cm. However, more sophisticated models are required to compute *R^p^* if *T*_B_*^p^* originates from a landscape, e.g., with vegetation.

Recent results obtained from several theoretical studies and field experiments dedicated to the retrieval of sea salinity as part of the SMOS mission are presented in [15]. For retrieving ocean salinity from *T*_B_*^p^* measured at L-band, the principle is similar as applied for retrieving soil moisture. Again *T*_B_*^p^* can be expressed by Equation (1). However, the dielectric constant of ocean water is in a quite different range. It is the imaginary part of the permittivity that increases with increasing salt content due to the increased conductivity. Sea salinity is measured in Practical Salinity Units (psu) defined as: Sea water with the salinity 35 psu has a conductivity ratio of unity at 15 °C (and 1 atmosphere pressure) with a potassium chloride (KCl) solution containing 32.4356 g of KCl per kg of solution. The salinity of the ocean is between 31 and 38 psu, but can be substantially less where mixing with fresh water occurs. The most saline open sea is the Red Sea (36–41 psu), but even higher values are found in isolated bodies of water, such as in the Dead Sea (300–400 psu). However, the sensitivity of *T*_B_*^p^* measured with respect to the salinity of the open ocean is approximately 1 K·psu^−1^ at vertical polarization and the observation angel of 50° relative to nadir.

### 1.2. SMOS Requirements

ESA’s SMOS mission, proposed in the framework of the Earth Explorer Opportunity Missions [16] aims at deducing soil surface moisture and ocean salinity with near global coverage every three days [17]. The mission’s requirements regarding soil moisture are: the accuracy should be better than 4% volumetric moisture with a spatial resolution of 35–50 km of a single measurement. The desired accuracy of ocean salinity retrieved from a single measurement is 0.5–1.5 psu. For a 30–day average over an area of 100 km × 100 km, the accuracy is specified to 0.1 psu, implying that brightness temperatures measured with the SMOS L-band radiometer have to be within ±0.1 K.

### 1.3. SMOS Calibration and Validation Activities

SMOS is the outcome of a long process initiated in late 1970s. During recent years, many research activities have been performed to support this mission (see [18] for an extensive overview of recent research activities related to SMOS). Many of these activities focused on questions concerning calibration and validation issues for soil moisture and sea salinity retrieval. Others were dedicated to the detection of biomass, or to technical aspects of the sensor. Regarding soil moisture retrieval, many experimental and theoretical studies have been performed to explore the radiative properties of the basic land-cover types considered in the so-called ‘L-band Microwave Emission of the Biosphere’ (L-MEB) model [19] which is the Level-2 algorithm to produce soil-moisture data. This research has mostly been performed with ground-based L-band radiometers either mounted on towers or cranes. Thus, a considerable number of L-band radiometers with sometimes different characteristics have been built [20] and operated by the scientific community.

Although our knowledge about the interaction between microwaves and land-surface features has increased dramatically in the course of these activities, further ground-based experiments during the SMOS commissioning and operative phases are essential. For this reason and to overcome the problem of different instrument performances affecting the L-band signatures, the construction of three identical L-band radiometers was recommended to ESA. These instruments have been built by a consortium consisting of an industrial partner (Metaplan, Adliswil, Switzerland) and two university partners (Institute of Applied Physics, Bern, Switzerland and Swiss Federal Research Institute WSL, Birmensdorf, Switzerland), headed by the company Gamma Remote Sensing AG (Gümligen, Switzerland), with a total budget of approximately 360 kEuro. The architecture of the three ELBARA II L-band radiometers is based on the ETH L-Band radiometer for soil-moisture research (ELBARA), [21] designed and built by the Institute of Applied Physics, University of Berne. This instrument has been successfully deployed in a series of field experiments [22–27]. However, major improvements of the microwave electronics, the mechanics, and the user interface have been made to the successor, ELBARA II. In particular the development of an Active Cold Source as an instrument internal calibration noise source has improved the absolute accuracy significantly.

## 2. Instrument Design

A microwave radiometer is a receiver for electromagnetic radiation with sub–millimeter to centimeter wavelengths, corresponding to the frequency range of 1–1,000 GHz. The L–band, ranging from 1–2 GHz, has many commercial and military applications. It also contains the hydrogen line at 1,420.41 MHz, originating from the hyperfine transition of neutral hydrogen. For the imaging of neutral atomic hydrogen in interstellar space, passive measurements at this frequency are of great astronomical interest. As a consequence, the 27 MHz frequency band ranging from 1,400 to 1,427 MHz has become a protected radio astronomy allocation world–wide, in which it is forbidden to transmit any kind of electromagnetic radiance. Likewise, an RFI–free environment is mandatory to measure microwave brightness temperatures emitted from terrestrial surfaces. The frequency transfer function of an L–band radiometer to be used for retrieving geophysical properties must therefore be narrow and within this protected band. This implies, however, that the power level *P* received by such a radiometer with bandwidth *B* = 27 MHz is very low. For a noise source at the physical temperature *T*, and with emissivity equal to unity (e.g., a perfectly matched resistor), the noise power received is:

(2) P=kTB

with *k* = 1.380658·10^−23^ J K^−1^ being the Boltzmann constant. The same expression holds true when a scene at the physical temperature *T* and with the emissivity 1 is observed with a radiometer. For *T* = 300 K, this gives *P* ≈ 0.11·10^−12^ W (≈−99.5 dBm) received with the radiometer antenna. To detect such an extremely low power, the radiometer (RM) must have the lowest possible residual noise *T*_RM 0_ and any instrument internal RFI disturbances must be rigorously mitigated. To allocate an absolute value to the noise power received with the antenna, the noise power must be compared with the power of at least two instrument internal calibration sources with known noise temperatures. Provided that the linearity of the receiver is sufficient and the gain is stable between several calibration cycles, this allows a certain brightness temperature to be assigned to the radiance entering the antenna. These requirements are important for the design of the ELBARA II electronics described below.

### 2.1. Block Diagram

**Figure 2.**
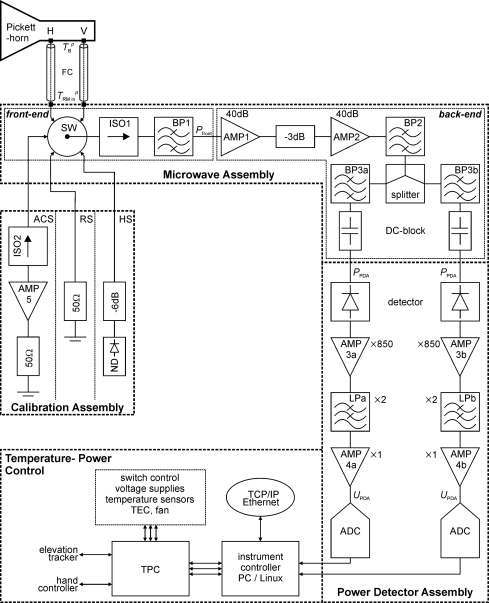
Block diagram of the ELBARA II radiometer. The abbreviations used and the specifications of the components are given in the Appendix.

The block diagram of the ELBARA II radiometer is shown in Figure 2, and the relevant specifications of the individual components are listed in the Appendix. The block diagram is subdivided into the sub-systems: Microwave Assembly, Power Detector Assembly, Calibration Assembly, and the Temperature–Power Control unit. The functionality of these sub–systems are outlined in Sections 2.1.1 to 2.1.4.

#### 2.1.1. Microwave Assembly

The Microwave Assembly (MA) consists of the components of the front-end and the back-end (Figure 2). The mechanical input switch (SW) allows the selection of the noise source fed to the MA input, which could be either one of the radiometer inputs *T*_RM,in_^H^ or *T*_RM,in_^V^ to measure antenna brightness at horizontal or vertical polarization, or one of the tree-internal reference noise sources. The output of the switch is fed through an isolator (ISO1) tuned to the center of the radiometer band at 1,413.5 MHz to ensures a good match of the selected noise input to the receiver path.

The microwave signal at the isolator output is directly fed into a 4-Section band-pass filter (BP1) before amplification. In this ELBARA II is unlike to other L-band radiometers currently used for the observation of terrestrial surfaces, e.g., the polarimetric radiometer EMIRAD [28] or the LEWIS radiometer [29]. The additional loss of <0.77 dB of the BP1 contributes less than 50 K to the total residual noise *T*_RM,0_ of the radiometer (see Section 2.2). This disadvantage is compensated for by the way RFI from outside the protected band is suppressed in the front-end before amplification, which avoids possible saturation of the first low-noise amplifier AMP1.

To keep the residual noise *T*_RM 0_ of the radiometer as low as possible, the total transmission loss *L*_front_ of the front-end must be minimized. This is due to the very low power level along the front-end, which is of the same order as the thermal noise caused by the losses of the front-end components. In accordance with (2), this is typically 10^−13^ W ≈ −100 dBm for the frequency and bandwidth considered. In the back-end, the power levels are approximately 77 dB higher, which implies that the corresponding losses do not impact *T*_RM, 0_ significantly.

The front-end components are the input noise-power switch (SW), the isolator (ISO1), the band-pass filter (BP1), and three semi-rigid coaxial cables (Huber Suhner, ≈12 cm) with SMA connectors. Assuming the loss of one cable as 0.1 dB and using the specifications given in the Appendix results in *L*_front_ ≈ 0.15 Db + 0.20 dB + 0.77 dB + 3·0.1 dB = 1.42 dB, corresponding to the power transmission factor *t*_front_ = 0.72. Where there is perfect matching (no reflection), this corresponds to the front-end absorptivity 1 − *t*_front_. Where the electronic components are in thermal equilibrium at the typical temperature *T*_0_ = 313 K (40 °C), the absorptivity equals the emissivity and the noise power caused by the front-end loss is *T*_0_·(1 − *t*_front_) ≈ 87 K. Besides the losses in the front-end, the performance of the first low-noise amplifier (AMP1) determines *T*_RM 0_. In accordance with its specified noise figure *NF* = 0.5 dB, the noise temperature *T*_AMP1_ of AMP1 is *T*_AMP1_ = *T*_0_·(10*^NF^*^/10^ − 1) ≈ 38 K. These considerations yield the residual noise of the ELBARA II radiometer estimated from the component specifications as *T*_RM 0_ = *T*_0_·(1 − *t*_front_) + *T*_AMP1_ ≈ 125 K for *T*_0_ = s313 K.

The output of AMP1 is attenuated by 3 dB and amplified a further 40 dB by AMP2. The output of AMP2 is filtered using a 6-Section band-pass filter. The band-pass filters (BP1/2) are both centered at 1,413.5 MHz and have a bandwidth of 22 MHz at −3 dB to be within the protected band allocation from 1,400 MHz to 1,427 MHz. The 3 dB attenuator between the amplifiers avoids amplifier instabilities.

The output of the second amplifier (AMP2) is split into two channels using a symmetric power splitter. The two outputs of the splitter are then filtered by 4-Section band-pass filters (BP3a/b) with the center frequencies 1,407.5 MHz and 1,419.5 MHz, respectively each with a −3 dB bandwidth of 11 MHz. In this way, two slightly overlapping receiver channels within the protected band are created, which allow narrow-band RFI to be detected within the protected band. The corresponding lower side band (LSB) and the upper side band (USB) of the MA back-end are AC-coupled to the detectors through DC-blocks in order to remove any low-frequency internal RFI or DC-bias signals from ground loops or pick-up from the radiometer electronics.

Summing up the specified losses (see Appendix) of the back-end components (attenuator (3 dB) + BP2 (1.22 dB) + splitter (0.4 dB) + one-to-one splitting into the LSB and the USB (3 dB) + BP3a/b (1.3 dB) + DC-block (0.15 dB) + five connecting cables (5·0.1 dB)) yields the loss *L*_back_ ≈ 9.57 dB of each of the two frequency channels. The MA gain *G*_MA_ ≈ 69.01 dB is estimated as the difference between the gain of the two low-noise amplifiers (2·40 dB) and the total loss *L*_MA_ = *L*_front_ + *L*_back_ = 10.99 dB of the MA.

Table 1 shows the typical noise temperatures applying at the MA inputs (column 1), the expected power levels *P*_front_ at the output of the front-end (column 2), and the associated power levels *P*_PDA_ (in units of dBm and μW) expected at the output of the back-end of the MA (columns 3 and 4). The selected MA inputs are: *T*_sky, in_ expected for a sky measurement; *T*_ACS_, *T*_RS_, and *T*_HS_ of the active cold source (ACS), the resistive source (RS), and the hot source (HS); and *T*_scene,min_, *T*_scene,max_ cover the range of land-surface brightness temperatures. The power levels *P*_front_ are derived as the sum of the noise power associated with the radiometer residual noise *T*_RM 0_ ≈ 125 K, plus the power due to the noise temperature applying at the MA input. Thereby, equation (2) is used with the bandwidth *B* = 22 MHz of the BP1. In units of dBm, the *P*_PDA_ are:

(3) $$P_{\text{PDA}}=P_{\text{front}}+G_{\text{MA}}$$

#### 2.1.2. Power Detector Assembly

The power detector assembly (PDA) depicted in the block diagram (Figure 2) determines the performance of the radiometer. The PDA is symmetrical in respect to the two frequency channels implemented. The LSB and the USB outputs of the MA are fed to Planar-Doped Barrier diode detectors that are terminated resistively with 10 kΩ for best linearity and minimum insensitivity to temperature variation. For the estimated input power range *P*_PDA_ (Table 1), the detectors operate well within their square-law regime. Therefore, the detector output voltage is directly proportional to *P*_PDA_ with a voltage sensitivity of >0.5 mV μW^−1^.

The detector output voltages are amplified using instrumentation amplifiers with voltage gains of approximately 850, and finally low-pass filtered with a cut-off frequency of 400 Hz at −3dB. Buffer amplifiers (AMP4a/b) are used to drive the 16-bit analog to digital converter (ADC), operating at the nominal sample rate of 1,600 Hz. However, sampling at 800 Hz is also feasible to reduce data volume and incurs only a small loss of radiometric sensitivity. After this point, processing is carried out by the on-board Instrument Controller (IC), which is part of the temperature and power control (TPC) unit described in Section 2.1.4.

#### 2.1.3. Calibration Assembly

**Figure 3.**
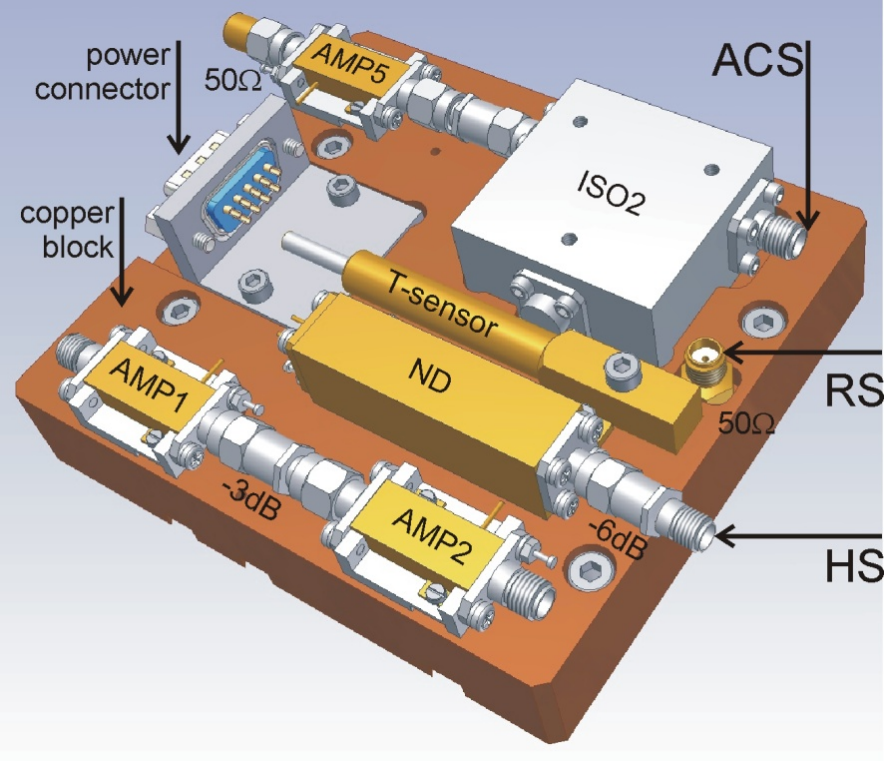
Layout of the calibration assembly (CA).

Internal calibration noise sources are used to determine the absolute values of noise temperatures, *T*_RM,in_^H^ and *T*_RM,in_^V^, applying at the radiometer input ports for horizontal and vertical polarization, respectively. As depicted in Figure 2, the input noise switch allows switching between *T*_RM,in_^H^ and *T*_RM,in_^V^ at the input ports and the internal calibration sources mounted on the Calibration Assembly (CA) depicted in Figure 3. The design is such that the losses between the radiometer input ports and the corresponding inputs of the switch are identical (≈0.05 dB). The same applies to the losses between the outputs of the three calibration sources and the inputs of the switch.

The CA consists of a heavy copper block (1.7 kg) on which the calibration sources and the two amplifiers (AMP1/2) used in the MA are mounted. A Thermo Electric Cooler (TEC) and a temperature sensor (T-sensor) are used for the thermal stabilization of the CA. This is crucial to maintain constant gains and noise added due to losses in the MA front-end. For the typical set point *T*_0_ = 313 K (40 °C), the temperature is measured to be within ±0.1 K. Furthermore, the CA is designed as a separate module to allow for independent operation for cross calibration among other L-band radiometers.

The resistive source (RS) consists of a standard 50 Ω SMA resistor tightly pressed into a borehole in the copper block to keep it constantly at the temperature *T*_0_. Accordingly, this results in the noise temperature *T*_RS_ = *T*_0_ of the RS equal to the CA temperature.

The hot source (HS) is made up of a commercial noise diode (ND) with the output attenuated by 6 dB. Given the noise temperature *T*_ND_ ≈ 1575 K of the factory-calibrated ND and the physical temperature *T*_0_ = 313 K of the −6 dB attenuator (with power transmission factor *t*_−6dB_ ≈ 0.251), the noise temperature *T*_HS_ of the HS is estimated as the transmitted part of *T*_ND_ plus the thermal noise of the attenuator. In case of perfect match between the components (no reflections) *T*_HS_ is:

(4) $$T_{\text{HS}}=T_{\text{ND}}\cdot t_{-6\text{dB}}+T_{0}\cdot (1-t_{-6\text{dB}})\approx 630\;K$$

The active cold source (ACS) is implemented with a low-noise amplifier (AMP5) with an isolator (ISO2) attached to the input and terminated with 50 Ω. The idea of this design is to use the low noise level of the amplifier (*T*_AMP5_ ≈ 34 K at *T*_0_ = 313 K) as a cold source. The isolator provides a good 50 Ω match between the ACS and the MA. In accordance with (4), the noise temperature *T*_ACS_ of the ACS is:

(5) $$T_{\text{ACS}}=T_{\text{AMP}5}\cdot t_{\text{ISO}2}+T_{0}\cdot (1-t_{\text{ISO}2})\approx 48\;K$$

The estimated *T*_ACS_ for *T*_0_ = 313 K is based on the component specifications and assumes perfect match between the components. However, *T*_ACS_ was determined more accurately by using *T*_RS_ and sky measurements (see Section 3.1.4) as reference sources.

#### 2.1.4. Temperature-Power Control

The ELBARA II instrument is controlled *via* two embedded computers. The temperature and power controller (TPC) is responsible for generating and monitoring the power used by the radiometer, controlling the Peltier heating and cooling element attached to the CA, and starting the main instrument computer (IC). The IC is responsible for scheduling system operations and acquiring data, communicating with the outside world, and controlling the radiometer-unit. The TPC is connected to the main IC *via* a serial line connection and acts after booting up the system as a slave to the IC. The IC polls the TPC for measurement values of the different temperature sensors attached to the TPC.

The total power dissipation by the radiometer electronics is about 45 W. The TPC uses a proportional-integral-derivative controller (PID) algorithm to adjust the current applied to a Peltier thermo-electric cooler (TEC) to ensure that the system temperature *T*_0_ is maintained at a constant value. Readings from the temperature sensors (precision of 0.003 K) positioned to monitor the actual CA temperature, the air temperatures inside and outside the electronics enclosure, and the heat sink temperature are used as inputs to the PID algorithm.

The TEC dissipates heat from the interior of the radiometer enclosure to the external environment *via* a heat sink attached to a fan controlled by the TPC. The TEC can also be used as a heat pump to heat the radiometer enclosure if necessary. If the heat sink is at the same temperature as the radiometer interior, the minimum cooling capacity of the TEC is 72 W. If the TEC is operated as a heat pump, it can generate up to 96 W. With this design, *T*_0_ can be maintained for the duration of the measurement cycle (≈30 seconds) within ±0.05 K of a fixed value, typically in the range of 10 K to 20 K above the external temperature.

### 2.2. Critical Components

#### 2.2.1. Feed Cables

The Pickett horn antenna features two ports, one for horizontal polarization (H) and one for vertical (V). As shown in Figure 2, the noise signals *T*_B_*^p^* (*p* = H, V) received are connected to the radiometer input ports, which can be switched *via* the input switch. The feed cables (FC) used are 0.3 m Huber+Suhner SUCOFLEX 106 coaxial cables with N-connectors on the antenna-side and SMA-connectors on the radiometer-side. These cables have losses of 0.26 dB·m^−1^ at 1.4 GHz. The transmission loss *L*_FC_ ≈ 0.1 dB (corresponding to the power transmission factor *t*_FC_ ≈ 0.98) of the FC is measured at ambient temperature and for the center frequency 1,413.5 MHz of the radiometer.

In analogy with (4) and (5), the noise temperatures at the radiometer inputs are *T*_RM in_*^p^* = *T*_B_*^p^*·*t*_FC_ + *T*_FC_·(1 − *t*_FC_), where *T*_FC_ is the temperature of the FC. The noise Δ*T*_B, FC_ = *T*_RM,in_*^p^* − *T*_B_*^p^* added by the FC is:

(6) $$\Delta T_{B,\text{FC}}=(1-t_{\text{FC}})(T_{\text{FC}}-T_{B}^{p})$$

This shows that the impact of the FC does not only depend on its loss, but also on the difference between *T*_B_*^p^* and *T*_FC_. In ground-based applications, the air temperature is a reasonable approximation for *T*_FC_, whereas *T*_B_*^p^* is generally smaller. In these cases, Δ*T*_B,FC_ is positive, implying that the thermal noise of the FC dominates its absorption. According to (6), Δ*T*_B,FC_ is largest for low *T*_B_*^p^* as measured with the instrument oriented towards the sky, and Δ*T*_B, FC_ decreases with increasing *T*_B_*^p^* (e.g., for *T*_FC_ = 300 K and *T*_B_*^p^* = *T*_sky_ ≈ 5 K ⇒ Δ*T*_B,FC_ = 6.7 K, and for *T*_B_*^p^* = 150 K ⇒ Δ*T*_B,FC_ = 3.4 K).

In Section 3, this simple model will be used for correcting the contribution Δ*T*_B,FC_ of the FC on the measurements *T*_RM,in_*^p^*. However, the model is not perfect because *T*_FC_, for example, is not constant along the FC. Therefore, Δ*T*_B,FC_ cannot be perfectly modeled, which makes it especially important to reduce the losses of the FC as far as possible.

#### 2.2.2. Input switch

Central to the radiometer operation is the electro-mechanical “Single Pole 6 Throw” input switch (SW). This precision RF switch (Agilent 87106B) is controlled *via* a TTL level signal to toggle between the different noise sources fed to the receiver path (Figure 2). As the switch is part of the MA front-end, it has to meet high demands in terms of its insertion loss *L*_SW_, repeatability, isolation and life-time. It is important for *L*_SW_ to be low and repeatable to minimize and control the noise added to the different inputs. High isolation is essential to prevent unwanted signals from interfering. For L-band frequencies, the maximum insertion loss is rated at *L*_SW_ = 0.15 dB for 10^7^ operations. The specified repeatability of the switch of 0.03 dB would imply that the residual noise *T*_RM 0_ can vary considerably (≈ 1.8 K). However, the repeatability measured was <0.005 dB (see Section 3.1.1) and therefore affects *T*_RM 0_ less than 0.3 K at *T*_0_ = 313 K. Hence, the switch performance is sufficient to function within the radiometer’s lifetime (at least 5 years), assuming that a full measurement cycle is performed every minute.

#### 2.2.3. Filters and Isolators

The insertion loss *L*_BP1_ of the 4-Section band-pass filter BP1 in the front-end of the MA contributes significantly to the residual noise *T*_RM 0_ of the radiometer, while the losses of the filters after the front-end are no longer critical. The selectivity and *L*_BP1_ of BP1 are coupled such that higher selectivity implies higher losses. The BP1 was selected to minimize *L*_BP1_, while maintaining acceptable selectivity outside of the protected band. To minimize the noise *T*_BP1_ of the BP1, a high quality silver-plated cavity filter was selected with rated *L*_BP1_ = 0.77 dB (corresponding to the power transmission factor of *t*_BP1_ = 0.84). For *T*_0_ = 313 K, this yields *T*_BP1_ = *T*_0_·(1 − *t*_BP1_) ≈ 50 K, which is 40% of the estimated *T*_RM 0_ = 125 K. For the same reason, ISO1/2 were tuned to have very low insertion losses *L*_ISO_ < 0.20 dB within the protected band (1,400 MHz–1,427 MHz), resulting in the relatively low noise *T*_ISO_ ≈ 14 K for *T*_0_ = 313 K.

#### 2.2.4. Amplifiers

Low-noise amplifiers AMP1/2/5 are selected to minimize the noises *T*_AMP1/2/5_ of the amplifiers in the low-signal parts of the radiometer (MA and ACS). Their noise figure is rated to *NF* < 0.5 dB over the protected band, corresponding to *T*_AMP1/2/5_ ≈ 34 K at *T*_0_ = 313 K. The instrumentation amplifiers AMP3a/b are selected to have a low input noise level, which contributes approximately 0.4 mV to the total uncertainty *σU*_RM_ of a single measurement *U*_RM_ performed with the shortest possible recording time *τ*_rec_ = 2.5 ms (see Section 3.2). Furthermore, the capability to easily set the gain with a single resistor was also considered in the selection.

### 2.3. Antenna

**Figure 4.**
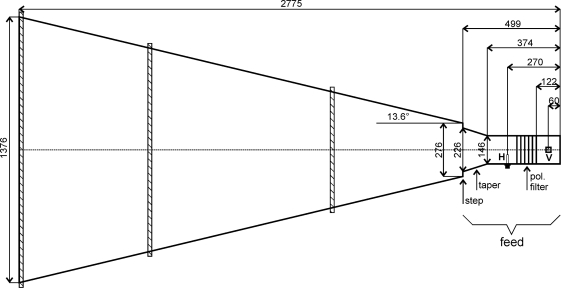
Sketch of the modified Pickett-horn antenna design. The dimensions relevant for the wave characteristics of the antenna are in units of millimeters.

The Pickett-horn antenna design shown in Figure 4 was selected for the ELBARA II system. This design has some modifications compared with the original Pickett-horn [30]: First, the diameter of the cylindrical waveguide of the antenna feed (the rear part) is smaller, and second, a taper acting as an additional phasing section has been introduced between this waveguide and the step transition. One characteristic of the original and the modified design is the highly directive far-field pattern with an expected −3 dB beam width of 12 (±6 around the antenna main direction). The antenna also provides horizontal and vertical polarization with symmetrical and identical beams with small side lobes.

The antenna feed, consisting of the cylindrical waveguide and the taper, is fabricated by turning from two heavy-walled aluminum tubes, resulting in an accuracy better than ±10 μm. The waveguide of the antenna feed comprises the two receiving *λ*/4-structures to receive the horizontally (H) and vertically (V) polarized radiance. To achieve a good isolation between the two orthogonal polarization directions (better than 40 dB; see Section 3.2.1.), 6 rods parallel to the horizontal polarization (pol. filter) are mounted in between. Each of the receiving *λ*/4-structures is made of a brass rod 5.25 mm in length, clamped to the interior-conductor of the N-connectors mounted in the waveguide. The lengths of the *λ*/4-structures are fine-tuned to minimize the return losses to achieve values smaller than −20 dB (see Section 3.2.1.).

The large antenna horn (the front part) screwed to the feed is produced from three rolled 3 mm aluminum sheets (PE100, semi-hard). The three cones 670 mm, 932 mm, and 670 mm in lengths are welded together and rigid aluminum rings (hatched) around the corresponding welding junctions and the aperture opening of the horn are attached. This measure increases the mechanical stability of the horn, as well as its rotational symmetry, which is of the order of ±1 mm. The two rings mounted at the joints are also used to attach the horn to the structure holding the antenna (see Figure 1 and Section 2.4). The ring at the antenna aperture can be used to mount auxiliary sensors, such as an infra-red radiometer or an optical camera, to observe the scene from the same observation angle as ELBARA II.

### 2.4. Scaffold and Elevation Tracker

The scaffold consists of a structure attached to the antenna horn (the antenna holder) and the suspension to mount the system either on a tower platform or on the cantilever of a crane (Figure 1). The construction is made of a space framework of rectangular hollow steel (EN 10219 S355J2H) sections welded together and hot-dip galvanized for corrosion protection. The cross beams with the most loads have cross-sections of 60 × 60 mm^2^ and thickness 3 mm, whereas the stabilizing cross beams have smaller dimensions (30 × 30 mm^2^).

The antenna holder is pivoted on the suspension which allows the antenna to be automatically angled to different elevations using a mechanical drive (elevation tracker). Elevation angles in the range 30° ≤ *α* ≤ 330° are supported (*α* = 180° is the zenith direction), enabling the observation of two diametrical footprints without rotating the instrument around its vertical axis. This is achieved by placing the suspension sufficiently high and by using a horseshoe-shaped base.

The elevation tracker comprises a two-stage worm gear (Atlanta, type BWS 58, reduction 1:39), attached to the antenna rotation axes and a planetary gear (Neugart, reduction 1:40) connected in series and propelled by an AC servo motor (JVL, type MAC141-A3AACA with MAC00-B4 extension module). This configuration results in the maximal mechanical torque of ≈1000 Nm, and features repeatable elevation positioning. The manufacturer of the gears rates the operational temperature range to be −20°C to +80°C.

The selected motor is equipped with an encoder that keeps the antenna at a constant orientation even under windy conditions. Furthermore, an inductive switch between the rotating part and the fixed scaffold is mounted to allow absolute positioning the antenna. The motor is powered and controlled through the embedded servo-drive, comprising an RS-232 interface that allows various state parameters also to be monitored, such as speed and torque. The motor conforms to IP67 and has a nominal operational temperature range of 0 °C to +40 °C, and a storage temperature range of −20°C to +85 °C. The electrical power consumption is 140 W at 48 V AC for 4,000 min^−1^. The entire system, including the scaffold, the elevation tracker, the antenna, and the radiometer electronics, weights approximately 500 kg.

### 2.5. Control of the Instrument

As discussed in Section 2 and illustrated in Figure 2, the instrument has two controllers, the Instrument Controller (IC) acting as master, and the Temperature Power Controller (TPC) acting as slave. The controllers communicate through two serial connections in master (IC)–slave (TPC) mode. The TPC is described in Section 2.1.4. In this section we will focus on the IC. The IC is based on a MSI GSE board with a low power Atom N270 processor running a stripped version of Ubuntu 9.04. Access to the IC is through an Ethernet (TCP/IP) connection. The selection of TCP/IP allows remote access to the instrument and has the advantage that various items that are available as shelf hardware can be built on, e.g., wireless links to the instrument. Two services for user interactions are running on the IC, a secure web server (lighttpd) and secure shell (openssh). The web server hosts an AJAX-enabled (PHP and Javascript) graphical user interface to operate the instrument, and can be accessed by any current web browser. The web interface enforces user authentication and communication is SSL encrypted. The following actions can be performed *via* the web interface: (i) accessing status information of the radiometer and of the elevation tracker; (ii) steering the elevation tracker; (iii) initiating ad-hoc measurements; (vi) managing files and maintaining the operating system (the full system is available through secure shell access); and (v) programming data acquisitions.

The selection of Free Open Source Software (FOSS) for the operating system, graphical user interface and instrument control (Python) has the advantage that additional functionality can easily be added to the instrument if necessary. For example a camera or additional sensors, such as an infrared radiometer, may be added. In addition to the instrument access through Ethernet, a hand-control interface can be used to start/stop the instrument, to show status information and to set some system parameters.

## 3. Instrument Characteristics and Tests

Section 3.1 presents the results from measurements performed on radiometer sub-systems. Section 3.2 focuses on the measured characteristics of the assembled ELBARA II system operated under field conditions.

### 3.1. Characteristics of Radiometer Sub-Systems

#### 3.1.1. Frequency Transfer Function of the Microwave Assembly

As already outlined in Section 2.1 and illustrated in the block diagram in Figure 2, the design of the MA and of the PDA comprise an LSB centered at 1,407.5 MHz and an USB centered at 1,419.5 MHz, both with 11 MHz bandwidth. This allows narrow band RFI occurring within the protected band 1,400 MHz to 1,427 MHz to be identified by monitoring differences between signatures at these two channels (frequency analyses).

**Figure 5.**
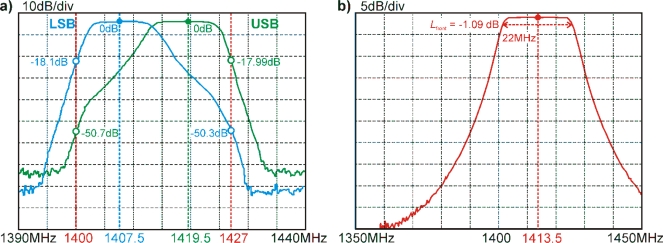
(a) Frequency transfer functions of the two frequency channels (LSB = blue, USB = green) of the microwave assembly. The borders of the protected part of the L-band at 1,400 MHz and 1,427 MHz are indicated in red. (b) Frequency response of the MA front-end. The loss measured at the radiometer center frequency of 1,413.5 MHz is *L*_front_ = 1.09 dB.

The measured frequency transfer functions of the two channels are shown in Figure 5a. The transfer function of the LSB (blue) is the result of the series connection of the band-pass filters BP1, BP2, and BP3a, whereas the transfer function of the USB (green) is determined by the characteristics of BP1, BP2 and BP3b. The critical attenuations at the limits of the protected band are −18.1 dB for the LSB and −17.99 dB for the USB, dropping off quickly to over −70 dB of attenuation within a few MHz.

#### 3.1.2. Front-End Loss

The total loss *L*_front_ of the front-end determines the residual noise *T*_RM 0_ of the radiometer. Based on the specifications (Appendx) of the front-end components (SW, ISO1, BP1, and three semi-rigid coaxial cables with SMA connectors) the total loss at the center frequency 1,314.5 MHz was estimated as *L*_front_ ≈ 1.42 dB, yielding *T*_RM 0_ ≈ 125 K for the system temperature *T*_0_ = 313 K (see Section 2.1.1). Figure 5b shows the measured frequency transfer function of the front-end. For the different switch inputs the measurements were within 0.015 dB, and the repeatability of consecutive measurements was better than the sensitivity of the measurements (≈0.005 dB).

The frequency response of the front-end is dominated by the characteristics of the BP1 with the specified −3 dB bandwidth of 22 MHz at the radiometer center frequency 1,314.5 MHz. At this frequency, the overall front-end loss measured is *L*_front_ ≈ 1.09 dB, which is well below the value expected from the specifications of the front-end components. Accordingly, the residual noise estimated from the measured *L*_front_ is *T*_RM 0_ ≈ 108 K, which is smaller than *T*_RM 0_ ≈ 125 K estimated using the component specifications.

#### 3.1.3. Linearity of the Power Detector Assembly

The PDA response is measured with the Micronetics noise module SNM 7114-C2A. Its output is band-pass filtered to cover the frequency range of 1,400 MHz–1,700 MHz, and then amplified by 30 dB yielding, a constant power level of *P*_0_ ≈ −25 dBm. Subsequently, *P*_0_ is passed through Agilent 9496B attenuators with the total attenuation variable in the range of 0 dB ≤ *A*_tot_ ≤ 20 Db with a step size of 1 dB.

For the attenuators set to *A*_tot_ = 0 dB, the power *P*_PDA_ ≈ −25 dBm injected to the PDA is measured with an Agilent power meter. This reference value and the selected *A*_tot_ is used to inject the power levels −25 dBm ≥ *P*_PDA_ ≥ −45 dBm (corresponding to 3.16 μW to 0.03 μW) into the detector diode of the PDA. The resulting output voltages *U*_PDA_ of the PDA are measured with a multimeter.

**Figure 6.**
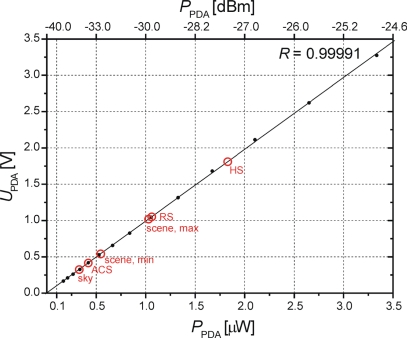
Measured voltage response *U*_PDA_(*P*_PDA_) of the PDA with respect to injected power in the range −38 dBm ≤ *P*_PDA_ ≤–25 dBm (black dots). The solid line is the linear fit (7) with the gradient *dP*_PDA_/*dU*_PDA_ = 1.00939 μW V^−1^. Red circles are the estimates of *P*_PDA_ for expected MA input noise temperatures (Table 1).

Figure 6 shows the response of the PDA with respect to input power levels −38 dBm ≤ *P*_PDA_ ≤ −25 dBm (0.17 μW ≤ *P*_PDA_ ≤ 3.33 μW), resulting in the range of output voltages 0.167 V ≤ *U*_PDA_ ≤ 3.334 V (black dots). The red circles are the *P*_PDA_ from Table 1 estimated for the MA input noise temperatures expected. Fitting the model *P*_PDA_ = *P*_PDA 0_ + *dP*_PDA_/*dU*_PDA_ · *U*_PDA_ to the measured relation between the power *P*_PDA_ [μW] and the voltages *U*_PDA_ [V] yields the small negative offset *P*_PDA 0_ ≈ −0.00352 μW with the standard error of ±0.00564 μW. Hence, an apparently negative offset *P*_PDA 0_ is not significant but explained by the errors in *P*_PDA_ caused by the known uncertainty (±0.05 dB) of the variable attenuator used in the test setup. Consequently, the PDA is virtually offset-free, implying that the simple linear model depicted in Figure 6 (solid line) is adequate:

(7) $$P_{\text{PDA}}\;[\mu W]=\frac{{\mathrm{dP}}_{\text{PDA}}}{{\mathrm{dU}}_{\text{PDA}}}\cdot U_{\text{PDA}}=1.00939\cdot U_{\text{PDA}}\;[V]$$

The asymptotic standard error of the power sensitivity *dP*_PDA_/*dU*_PDA_ = 1.00939 μW V^−1^ is ±0.00247 μW V^−1^. This and the correlation coefficient *R* = 0.99991 confirms the highly linear response of the PDA within a power range that includes the power levels estimated for the operative mode of the instrument.

#### 3.1.4. Characteristics of the Active Cold Source

In Section 2.1.3, the ACS noise temperature is estimated to *T*_ACS_ ≈ 48 K, based on the specifications (Appendix) of the components involved (Figure 2, ISO2 and AMP5) at the physical temperature *T*_0_ = 313 K. The low *T*_ACS_ makes it challenging to calibrate the ACS absolutely in a lab experiment. On the one hand, the impact of losses is strong and difficult to control and, on the other, it is difficult to find a highly accurate noise standard with an even lower noise temperature.

Nevertheless, such lab measurements are performed using the resistive source (RS) at *T*_RS_ = 300 K and the calibrated noise diode at *T*_ND_ = 1575 K as standards to be compared with *T*_ACS_, which is to be determined. After amplifying these noise temperatures with the two amplifiers of the MA, their frequency responses are measured with a Agilent E4408B spectrum analyzer. The associated power levels for the frequency range (1,413 ± 500) MHz are determined to be *P*_ACS_ = 0.778 μW, *P*_RS_ = 2.748 μW, and *P*_HS_ = 11.888 μW. Finally, the known reference noise temperatures *T*_RS_ = 300 K and *T*_ND_ = 1,575 K are used to determine *T*_ACS_ ≈ 39 K by considering a linear relation between power and injected noise.

This calibration of the ACS is error-prone due to the applied extrapolation, which multiplies the measurement uncertainties of the reference sources. Hence, a calibration procedure using the RS and the cold sky as a reference source is applied to determine *T*_ACS_ more accurately. The noise temperature of the RS is *T*_RS_ = *T*_0_, which is significantly higher than *T*_ACS_. In contrast, the noise standard *T*_sky, in_ = *T*_sky_ + Δ*T*_B, FC_ ≈ 10 K (see Section 2) at the input of the radiometer looking towards the sky is smaller than *T*_ACS_, which allows the ACS to be calibrated using linear interpolation instead of extrapolation:

(8) $$T_{\text{ACS}}=\frac{T_{\text{RS}}-T_{\text{sky},\text{in}}}{U_{\text{RS}}-U_{\text{sky},\text{in}}}\left(U_{\text{ACS}}-U_{\text{sky},\text{in}}\right)+T_{\text{sky},\text{in}}$$

The output voltage *U*_RS_ is measured for the resistive source (RS) switched to the radiometer input port, and *U*_sky, in_ is measured with the instrument oriented towards the sky. As described in Section 2, *T*_sky, in_ = *T*_sky_ + Δ*T*_B, FC_ is the received sky brightness *T*_sky_, complemented with Δ*T*_B, FC_ due to the loss of the FC. According to (6), the latter is particularly significant for low antenna brightness such as *T*_sky_. If the radiometer is not pointing exactly towards the galaxy, the sun, or the moon, *T*_sky_ received varies marginally over the sky hemisphere. In this case *T*_sky_ can be computed as the sum of the down-welling atmospheric radiance plus the cosmic background emission (assumed to be 2.7 K), attenuated by the atmosphere [31]. Evaluating the model [31] for the radiometer set-up at WSL (zenith angle *θ* = 30, elevation 554 m a.s.l.) and air temperatures between 0 °C and 30 °C yielded 4.44 K ≤ *T*_sky_ ≤ 4.48 K.

These theoretical values are used in the calibration procedure to determine *T*_ACS_ for 7 different set point temperatures *T*_0_ of the assembled ELBARA II system (Figure 1). The data to determine *T*_ACS_ consist of records of single radiometer voltages *U*_sky in_, *U*_RS_, and *U*_ACS_ measured every 10 minutes between 10 p.m. and 2 a.m. on seven successive days in April 2009. This time period is selected in order to avoid disturbances caused by the galaxy passing through the field of view and to ensure the atmospheric conditions are comparable every day. The measurements are performed for *T*_0_ in the range of 21 °C–39 °C in steps of 3 °C. To give the temperature feedback loop sufficient time to settle before the next measurements, *T*_0_ is switched to the next higher value several hours before measuring.

The voltages are recorded with the ADC sampling rate of *f*_ADC_ = 800 Hz, which is twice the nominal −3 dB cut-off frequency of the low-pass filters LPa/b in front of the ADC. This implies that almost the maximum time resolution of nominally 2.5 ms of the radiometer output voltage is recorded. For the duration of data recording set to 10 seconds, this corresponds to the nominal total integration time of 10 seconds per voltage data record. As will be discussed in Section 3.2 (Table 2b), these settings yield data records with mean values 〈*U*_sky, in_〉, 〈*U*_RS_〉, 〈*U*_ACS_〉 and very small standard deviations *σU*_sky, in_, *σU*_RS_, *σU*_ACS_.

**Figure 7.**
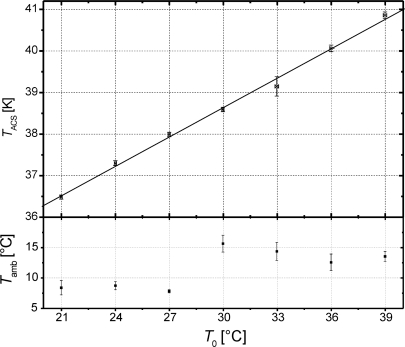
Mean noise temperatures *T*_ACS_ of the ACS measured for instrument set-point temperatures *T*_0_ = 21 °C, 24 °C, 27 °C, 30 °C, 33 °C, 36 °C, 39 °C (top panel), and ambient temperatures *T*_amb_ measured during the calibration measurements (bottom panel).

The crosses in the top panel of Figure 7 are the mean *T*_ACS_ derived for a specific *T*_0_. The error bars indicate the associated standard deviations *σT*_ACS_ ≤ 0.23 K and *σT*_0_ ≤ 0.1 K. This shows that the temperature feedback loop is able to keep *T*_0_ very stable for the ranges of ambient temperatures *T*_amb_ indicated in the bottom panel of Figure 7. The variation *σT*_ACS_ is less than 1% of the measured mean *T*_ACS_. It is assumed that most of these variations are due to errors introduced by the model (6), which uses *T*_amb_ to correct for the noise contribution Δ*T*_B, FC_ of the FC to *T*_sky_.

The solid line in Figure 7 is the linear interpolation to the mean *T*_ACS_:

(9) $$T_{\mathrm{ACS}}\;[K]=T_{\mathrm{ACS},{}^{\circ}C}+\frac{{\mathrm{dT}}_{\mathrm{ACS}}}{{\mathrm{dT}}_{0}}\cdot T_{0}=31.56353K+0.23579\cdot T_{0}[{}^{\circ}C]$$

The positive temperature response *dT*_ACS_/*dT*_0_ ≈ 0.24 K°C^−1^ is mostly associated with the noise caused by the loss of the isolator (ISO2), which increases with *T*_0_ (Figure 2).

The calibration procedure based on sky and RS measurements is also applied to the HS. The measured noise temperatures of the HS for 21 °C ≤ *T*_0_ ≤ 39 °C are in the range of 630 K < *T*_HS_ < 660 K, which is in good agreement with the estimated value given in Table 1. The temperature response *dT*_HS_/*dT*_0_ ≈ 1.60 K °C^−1^ measured is highly linear for the temperature range considered and mostly due to the increasing noise of the 6 dB attenuator attached to the output of the ND (Figure 2).

### 3.2. ELBARA II Characteristics

#### 

The radiometer output voltages, *U*_RS_ and *U*_ACS_, recorded for the two frequency channels with the RS and the ACS switched to the MA are used to determine the most important system parameters. The same settings as these given in Section 3.1.4 applied to calibrate the ACS are used (sampling with *f*_ADC_ = 800 Hz during 10 seconds). The data set used consists of measurements performed every 10 minutes for 4 hours, resulting in a total of 2·24 data records for the RS and the ACS.

The characteristics measured for *T*_0_ = 313 K (40 °C) are summarized in Tables 2 and 3. The parameters measured are: The radiometer gain *G*_RM_, the residual noise temperature *T*_RM 0_, the time bandwidth product *Bτ* of a single measurement associated with the smallest possible integration time (nominally 2.5 ms), the voltage noise *σU*_PDA_ of the PDA, the overall accuracies *σU*_RM_ of radiometer output voltages, and the corresponding accuracies *σT*_B_ of brightness temperatures measured.

The equations used to derive *G*_RM_, *T*_RM 0_, *Bτ*, *σU*_PDA_, and *σU*_RM_, *σT*_B_ from the measurements *U*_RS_ and *U*_ACS_ are discussed below. The following conventions are used: (i) Voltages averaged over one data record of 10 seconds duration are 〈*U*_RS_〉, 〈*U*_ACS_〉 and their standard deviations are *σU*_ACS_, *σU*_RS_; (ii) the noise temperature of the RS is *T*_RS_ = *T*_0_ (measured once for each data record); (iii) the expression (9) evaluated for measured *T*_0_ is used for *T*_ACS_; (vi) the values given in Table 2 are mean values derived from the 2·24 data records *U*_RS_, *U*_ACS_.

##### Gain

The radiometer gain *G*_RM_ = *dU*_RM_*/dT*_RM, in_ measures the response *dU*_RM_ of the radiometer output voltage *U*_RM_ with respect to a change *dT*_RM, in_ in the input brightness temperature *T*_RM, in_. If the system response is considered linear, *G*_RM_ is:

(10) $$G_{\text{RM}}=\frac{\langle U_{\text{RS}}\rangle -\langle U_{\text{ACS}}\rangle }{T_{\text{RS}}-T_{\text{ACS}}}$$

The radiometer gains derived for the LSB and the USB channel differ by approximately 7%. This can be explained as due to small differences in the losses and gains of the components after the power splitter (Figure 2). However, the standard deviations of the *G*_RM_ are very small for both channels. This is important to note, as it implies that *G*_RM_ is highly stable during a measurement cycle, which lasts less than a minute, where the internal calibration and antenna are measured.

##### Residual noise

The linear extrapolation of the relation *T*_RM, in_(*U*_RM_) to the value *U*_RM_ = 0 V yields the radiometer residual noise temperature:

(11) $$T_{\text{RM},0}=\frac{\langle U_{\text{RS}}\rangle }{G_{\text{RM}}}-T_{\text{RS}}$$

As outlined in Section 2.1.1, *T*_RM, 0_ is mainly due to the noise of the first amplifier (AMP1) in the MA and to the loss along the front-end. As these microwave components are common for the two channels (Figure 2), the *T*_RM, 0_ for the LSB and the USB channels tend to be similar. However, *T*_RM,0_ ≈ 153 K given in Table 2 is larger than the value *T*_RM,0_ ≈ 125 K estimated from the component specifications, and also larger than *T*_RM,0_ ≈ 108 K estimated from the measured front-end loss. This is most likely due to small mismatches between the front-end components (SW, ISO1, BP1) causing reflections not considered in the estimation of *T*_RM,0_. Furthermore, a higher noise figure for the amplifier as a result of the higher internal physical temperature could explain the difference.

##### Time bandwidth product

The uncertainty *σU*_RM_ of the radiometer output voltage *U*_RM_, depends on the product *Bτ* of the radiometer effective bandwidth *B* and the effective integration time *τ* used to measure *T*_RM, in_. The low frequency noise *σU*_PDA_ of the PDA may also contribute to *σU*_RM_. Assuming these two voltage noise contributions are quasi-Gaussian and uncorrelated, the variance *σU*_RM_^2^ can then be expressed as:

(12) $${\sigma U}_{\text{RM}}^{2}=\frac{G_{\text{RM}}^{2}{\left(T_{\text{RM},\text{in}}+T_{\text{RM},0}\right)}^{2}}{B\tau }+{\sigma U}_{\text{PDA}}^{2}$$

Provided that measurements *σU*_RM_ = *σU*_ACS_ and *σU*_RM_ = *σU*_RS_ for the two different *T*_RM,in_ = *T*_ACS_ and *T*_RM,in_ = *T*_RS_ are available, *Bτ* and *σU*_PDA_ can be derived by solving the corresponding two equations of the form (12):

(13) $$B\tau =\frac{G_{\text{RM}}^{2}\;\left[{\left(T_{\text{RS}}+T_{\text{RM},0}\right)}^{2}-{\left(T_{\text{ACS}}+T_{\text{RM},0}\right)}^{2}\right]}{{\sigma U}_{\text{RS}}^{2}-{\sigma U}_{\text{ACS}}^{2}}\;\;\;\text{and}\;\;\;{\sigma U}_{\text{PDA}}^{2}={\sigma U}_{\text{RS}}^{2}-\frac{G_{\text{RM}}^{2}\;{\left(T_{\text{RS}}+T_{\text{RM},0}\right)}^{2}}{B\tau }$$

As can be seen in Table 2, the relative difference between *Bτ* found for the LSB and the USB are very small (≈0.5%), whereas the *σU*_PDA_ of the two PDA channels differ significantly. Measurements on the PDA alone revealed *σU*_PDA_ < 1 mV, mostly generated by the instrumentation amplifiers (≤0.6 mV), but also by noise leakage coupled e.g., through the power supply (≤0.3 mV). These measurements are in accordance with *σU*_PDA_ given in Table 2. They also explain the significant difference between the USB and the LSB frequency channels.

Considering the nominal values for the bandwidth (11 MHz) and the integration time (2.5 ms), the nominal time bandwidth product would be *Bτ* = 27,500 Hz s, which is significantly larger than *Bτ* actually measured (Table 2). However, this is to be expected as neither the frequency transfer function of a channel (Figure 5a) nor the post detection frequency cut-off of *f*_LP_ = 400 Hz (LPa/b) are ideal, which means that the real filter characteristics are not step functions at their band edges, but rather −3 dB values. This implies that the effective channel bandwidth, as well as the effective integration time, are both smaller than the nominal values. However, it is not critical to know the effective bandwidth and integration time precisely since the measured *Bτ* determine the measurement uncertainty.

Table 3 shows uncertainties *σU*_RM_ for three noise temperatures *T*_in, RM_ = 10 K, 41 K, 313 K at the MA input, corresponding to the approximate values for a sky measurement, the ACS and the RS noise. The *σU*_RM_ for *τ*_rec_ = 2.5 ms are uncertainties of single measurements *U*_RM_ with the shortest possible integration time (<2.5 ms), limited by the applied post-process low-pass filtering (*f*_LP_ = 400 Hz). The corresponding values are computed with (12) using the parameters *G*_RM_, *T*_RM 0_, *Bτ*, *σU*_PDA_ measured (right column in Table 2). The values shown in Table 3 agree well with the standard deviations of all the voltages, *U*_RM_ and *U*_ACS_, measured (*σU*_RM_ = 6.912 mV and *σU*_ACS_ = 2.924 mV). Furthermore, the distribution of these voltages closely follows a Gaussian distribution.

The *σU*_RM_ for *τ*_rec_ = 1 s, 3 s, 10 s given in Table 3 are computed from *σU*_RM_ for *τ*_rec_ = 2.5 ms by considering that the standard deviation decays with *N*^−1/2^, where *N* = *f*_LP_·*τ*_rec_ is approximately the number of independent measurements available in a data record. This is the consequence of sampling the low-pass filtered signal with the −3 dB cut-off frequency *f*_LP_ = 400 Hz with *f*_ADC_ = 2· *f*_LP_ = 800 Hz.

##### Accuracy of brightness temperatures

The uncertainty *σT*_B_ of a brightness temperature *T*_B_ measured is proportional to the uncertainty *σU*_RM_ scaled with the radiometer gain *G*_RM_:

(14) $${\sigma T}_{B}=\frac{{\sigma U}_{\text{RM}}}{G_{\text{RM}}}$$

The uncertainties *σT*_B_ given in the right three columns of Table 3 are expected for the indicated *T*_in, RM_ and *τ*_rec_. The uncertainties *σT*_B_ for *τ*_rec_ ≥ 3 s become smaller than 0.1 K for all input brightness temperatures that can be expected in applications of the radiometer. Therefore, a record duration of *τ*_rec_ = 3 s, is recommended for operating ELBARA II.

#### 3.2.1. Antenna

The return loss and the isolation between the horizontal and the vertical port of the antenna are important parameters. Both are measured with an Agilent E4408 spectrum analyzer attached to the antenna pointed towards the sky. Furthermore, knowing the directivity of the horn antenna is essential to know, as it determines the extent of the observed footprint. Measurements of these antenna characteristics are presented hereafter.

##### Return loss

**Figure 8.**
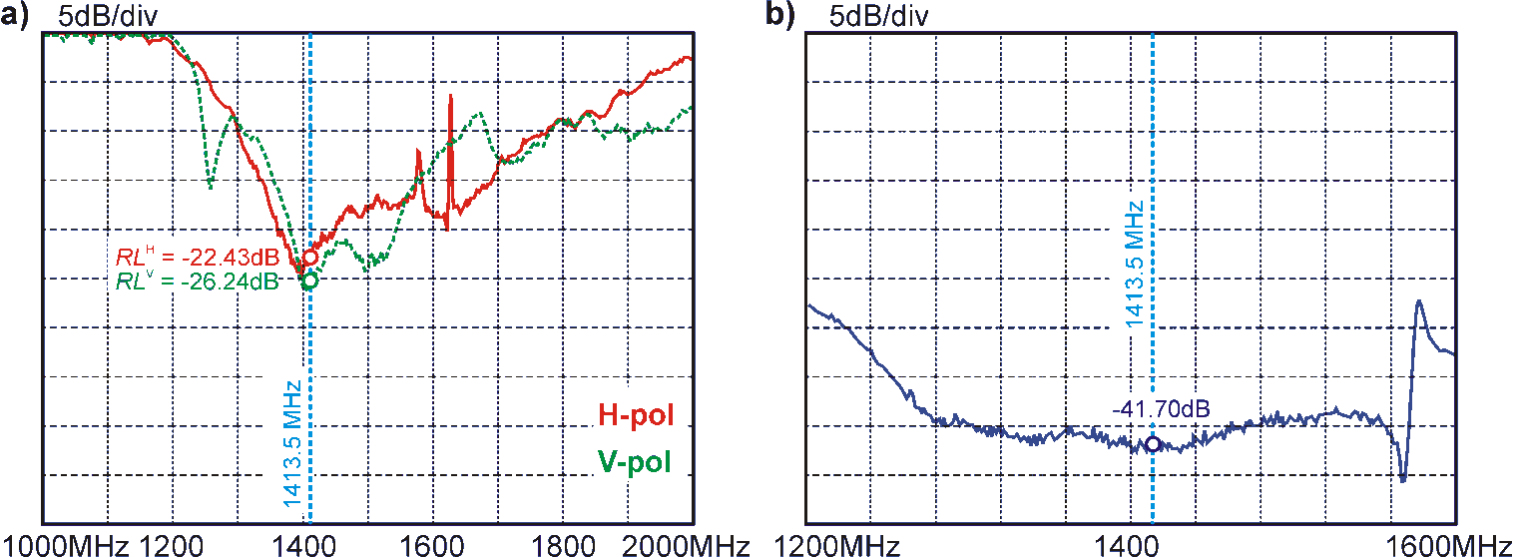
(**a**) Return losses *RL^p^* of the antenna feed measured after fine-tuning the receiving *λ*/4-elements for horizontal (red solid) and vertical (green dashed) polarization. (**b**) Measured isolation between the H- and the V port of the antenna. The radiometer center frequency at 1,413.5 MHz is indicated.

Figure 8a shows return losses *RL^p^* measured for the two ports (*p* = H, V) of the antenna. Measurements are performed for the frequency range of 1,000 MHz to 2,000 MHz using a spectrum analyzer and a directional coupler with directivity of about 20 dB–30 dB. The *RL^p^* [dB] shown are achieved after fine-tuning the length of the *λ*/4-structures receiving the radiances at H- and V-polarization, respectively (see Section 2.3). The measurements show that the specified value of *RL^p^* ≤ −20 dB are well met for the radiometer center frequency of 1,413.5 MHz.

##### H-V isolation

The isolation between the H- and V ports of the antenna (Figure 8b) was measured for 1,200 MHz to 1,600 MHz with the spectrum analyzer featuring an internal tracking source. The isolation is relatively constant over the radiometer bandwidth (1,400 MHz–1,427 MHz) and has a value of −41.7 dB at the radiometer center frequency. The measurements were the same, for either choosing the H- or the V port as the source. For a brightness temperature *T*_B_*^p^* = 300 K this implies that the distortion caused by polarization crosstalk is less than 0.025 K, and therefore negligible.

##### Directivity

The directivity of the rotation-symmetric Pickett-horn antenna described in Section 2.3 is derived from polarization averaged brightness temperatures *T*_B_ = (*T*_B_^H^ + *T*_B_^V^)/2, measured with the radiometer looking towards the sky. Brightness temperatures *T*_B_*^p^* = *T*_RM in_*^p^* − Δ*T*_B, FC_ entering the antenna aperture are deduced from *T^p^*_RM in_ (*p* = H, V) at the two radiometer input ports, corrected for the noise contribution Δ*T*_B, FC_ of the FC computed with (6) (*t*_FC_ = 0.98 corresponding to *L*_FC_ ≈ 0.1 dB):

(15) $$T_{B}^{p}=\frac{T_{\text{RM},\text{in}}^{p}-(1-t_{\text{FC}})T_{\text{FC}}}{t_{\text{FC}}}$$

Data records, *U*_RS_, *U*_ACS_, *U*^H^, *U*^V^, with duration *τ*_rec_ = 5 s acquired with *f*_ADC_ = 800 Hz and the set point *T*_0_ = 305 K are measured every 10 minutes. The actual temperatures, *T*_0_ and *T*_FC_ (FC temperature ≈ air temperature), are measured for each record. Furthermore, *T*_RS_ = *T*_0_ = 32 °C (305 K) is assumed, and *T*_ACS_(*T*_0_) is determined with (9). Based on these data records, the noise temperatures *T^p^*_RM in_ at the two radiometer input ports are derived as:

(16) $$T_{\text{RM},\text{in}}^{p}=\frac{\langle T_{\text{RS}}\rangle -\langle T_{\text{ACS}}\rangle }{\langle U_{\text{RS}}\rangle -\langle U_{\text{ACS}}\rangle }\left(U^{p}-\langle U_{\text{ACS}}\rangle \right)+\langle T_{\text{ACS}}\rangle$$

**Figure 9.**
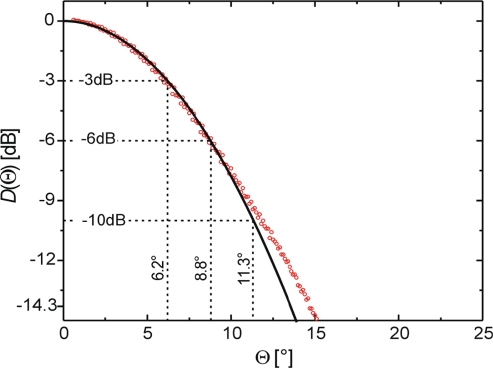
Normalized antenna directivity (red circles) derived from time series of brightness temperatures *T*_B_ measured with the sun passing through the antenna field of view. The solid line is the fitted Gaussian bell-shaped curve (17).

Measurements *T*_B_ = (*T*_B_^H^ + *T*_B_^V^)/2 were taken every 10 minutes between 11 a.m. and 4 p.m. on 30 April 2009. The antenna was oriented towards the South at the zenith angle *θ* ≈ 58°, such that the sun was in the main observation direction at 1:25 p.m. At that time, the maximum brightness measured was *T*_sun_ ≈ 180.8 K. To relate the times from the sky measurements, the polar angles Θ between the main direction of the antenna and the sun’s position at that time was also computed using [32]. This provides a relation *T*_B_(Θ), which is used to derive the normalized antenna directivity *D*(Θ) shown in Figure 9.

This is obtained by first subtracting the base line value *T*_B_(Θ > 30°) ≈ *T*_sky_ ≈ 5 K from *T*_B_(Θ), and then normalizing the resulting values to unity for Θ = 0°. The normalized directivity (red circles) measured and the fitted Gaussian bell-shaped model *D*(Θ) with Θ in units of degrees (solid black line) are shown.

(17) $$D(\Theta )=\text{exp}(-0.01781\cdot \Theta^{2})$$

The sensitivity of the measured *D*(Θ) is limited by the contrast *T*_sky_ / *T*_sun_ ≈ −14.3 dB. Therefore, the measurements are considered to be reliable for Θ ≤ 15°, implying that side lobes with *D* < −14.3 dB are not identified by the chosen measurement approach.

The beam widths at *D* = −3 dB, −6 dB, and −10 dB derived with (17) are at *θ* ≈ 6.2°, 8.8°, and 11.3°. Strictly speaking the solar disc is not a point source but exhibits a full angle of ≈ 0.5° at L-band frequencies. This implies that the measured *D*(Θ) is the convolution of the antenna directivity with the solar disc. Nevertheless, the measured characteristic angles agree very well with the numerical simulations [30], yielding ≈ 6° at −3 dB, 9° at −6 dB, and 12° at −10 dB.

## 4. Final Remarks

The production of the tree ELBARA II systems was completed in May 2009. The project ended and was accepted after being successfully reviewed by the ESA authorities after a workshop dedicated to the selected users of the instruments. During this workshop, held at WSL Birmensdorf, the operation of the system was explained and demonstrated to the users selected by ESA, which owns the radiometers. Shipping the instruments to the two SMOS core validation sites (Valencia anchor station (Spain) and University of Munich, Germany) and to the Finnish Meteorological Institute in Sodankylä took place in August 2009 after last improvements on the electronics had been made. The ELBARA II radiometers were mounted in schedule on the dedicated field sites before the launch of the SMOS satellite on 2 November 2009.

## Figures and Tables

**Table 1.** Power levels *P*_front_ at the output of the MA front-end and *P*_PDA_ fed to the PDA for typical input noise temperatures at the MA. PDA output voltages *U*_PDA_, considering the measured PDA sensitivity (7), are shown in the last column.

**MA input noise [K]**	***P*_front_ [dBm]**	***P*_PDA_ [dBm]**	***P*_PDA_ [μW]**	***U*_PDA_ [V]**

*T*_sky, in_ = 10	−103.8	−34.8	0.328	0.324
*T*_ACS_ = 48	−102.8	−33.8	0.420	0.416
*T*_scene, min_ = 100	−101.6	−32.6	0.545	0.540
*T*_scene, max_ = 300	−98.9	−29.9	1.029	1.019
*T*_RS_ = 313	−98.8	−29.7	1.060	1.050
*T*_HS_ = 630	−96.4	−27.4	1.827	1.810

**Table 2.** System parameters of the ELBARA II radiometer measured at *T*_0_ = 313 K (40 °C).

**System Parameter**	**LSB channel**	**USB channel**	**both channels**

*G*_RM_ [mV K^−1^]	1.93 ± 0.01	1.79 ± 0.01	1.86
*T*_RM, 0_ [K]	147.0 ± 0.3	158.8 ± 0.3	153
*Bτ* [Hz s]	15,908 ± 291	15,828 ± 271	15,868
*σU*_PDA_ [mV]	0.849 ± 0.150	0.449 ± 0.093	0.649

**Table 3.** Estimated uncertainties *σU*_RM_ and *σT*_B_ of measured radiometer voltages *U*_RM_ and brightness *T*_B_ for several radiometer inputs *T*_in, RM_ and durations *τ*_rec_ of the recorded data (*T*_0_ = 313 K).

*τ*_rec_ [s]	***σU*_RM_ [mV] for several *T*_RM, in_**			***σT*_B_ [K] for several *T*_RM, in_**		

	**10 K (sky)**	**41 K (ACS)**	**313 K (RS)**	**10 K (sky)**	**41 K (ACS)**	**313 K (RS)**

2.5·10^−3^	2.493	2.937	6.911	1.34	1.58	3.72
1	0.125	0.147	0.346	0.07	0.08	0.19
3	0.072	0.085	0.199	0.04	0.05	0.11
10	0.039	0.046	0.109	0.02	0.02	0.06

## Abbreviations

**Table:**

**Symbol**	**Meaning**
ACS	Active Cold Source
ADC	Analog-to-Digital Converter
AMP	AMPlifier
BP	Band Pass filer
CA	Calibration Assembly
ELBARA	ETH L-Band Radiometer for soil-moisture research.
ESA	European Space Agency
FC	Feed Cable
HS	Hot Source
IC	Instrument Controller
ISO	ISOlator
LP	Low Pass filer
MA	Microwave Assembly
ND	Noise Diode
PDA	Power Detector Assembly
PDU	Power Distribution Unit
PID	Proportional–Integral–Derivative controller
RFI	Radio Frequency Interferences
RM	RadioMeter
RS	Resistive Source
SMOS	Soil Moisture and Ocean Salinity mission
SW	input SWitch
TEC	Thermo-Electric Cooler
TPC	Temperature and Power Controller
USB / LSB	Upper / Lower Side Band

## Appendix

Specifications of the Electronic Components Used

**Table:**

**Component**	**Specifications**	**Functionality**
SW	Insertion loss < 0.1 dB Repeatability < 0.03 dB Mechanical switch >10^7^ operations 4-bit TTL control	Switching between H-, V-polarization and the calibration sources.
ISO1, ISO2	Tuned frequency = 1,413.5 MHz Loss = 0.20 dB	Improve matching of amplifier inputs.
BP1	Center frequency = 1,413.5 MHz Insertion loss < 0.77 dB −3 dB bandwidth = 22 MHz Min. attenuation at 1,391 MHz = 12 dB	Suppression of out-of-band RFI before amplification. Determines the radiometer frequency response.
BP2	Center frequency = 1,413.5 MHz Insertion loss < 1.22 dB −3 dB bandwidth = 22 MHz Min. attenuation at 1,391 MHz = 24 dB	Further suppression of out-of-band RFI. Determines the radiometer frequency response.
BP3a, (BP3b)	Center frequency = 1,407.5 MHz (1,419.5 MHz) Insertion loss < 1.3 dB −3 dB bandwidth = 11 MHz Min. attenuation at 1,391 MHz = 21 dB	Split the noise power into 2 spectral bands for RFI detection in the frequency domain.
LPa/b	Cut-off frequency = 400 Hz *G* = 2 (active filter) 4^th^ order LPF	Amplification and filtering of the detector output.
AMP1, AMP2, AMP5,	*G* = 40 dB *NF* = 0.5 dB (*T*_AMP_ =34 K at *T*_0_ = 313 K)	Amplify noise power (AMP1/2) and act as cold noise source (AMP5).
AMP3a/b	DC-instrumentation amplifier Gain = 850 Noise = 8 nV·Hz^−1/2^ Offset < 25 μV	Amplification of detector output voltage.
AMP4a/b	Buffer amplifier Gain = 1 Offset = < 10 mV	Drive for ADC input.
splitter	Insertion loss < 0.4 dB at 1,000 − 2,000 MHz Amplitude imbalance < 0.01 dB.	Splitting RF into two channels for RFI detection in the frequency domain.
DC-block	Insertion loss < 0.15 dB	remove low-frequency internal RFI or DC-bias signals.
detector	Zero-bias detector diode Low-level sensitivity = 0.4 mV/μW Noise < 50 μV Frequency range: 0.01–12 GHz	Square-law detection of the RF-power.
ADC	Number of channels = 4 Resolution = 16 bit Input voltage range = ±2.5V	Digitize the amplified, filtered, and detected noise.
source	Noise temperature ≈ 1,575 K	Additional hot calibration source.
